# The effect of wearable technology on psychomotor agitation in patients with diagnostic patients with schizophrenia expansion and psychosis

**DOI:** 10.1192/j.eurpsy.2023.1049

**Published:** 2023-07-19

**Authors:** F. Oflaz, P. Cetinay Aydin, T. Sahin Tokatlioglu, E. Eser, N. Zemen, B. Semiz, Y. Z. Hayirlioglu

**Affiliations:** 1 Koç University; 2 Bakırköy Mazhar Osman Mental Health and Neurological Diseases Education and Research Hospital; 3 Istanbul Aydın University, Istanbul, Türkiye

## Abstract

**Introduction:**

Due to the exacerbation of psychotic processes in acute psychiatric services, patients may exhibit risky behaviors for themselves and others. Especially, psycho-motor agitation seen in schizophrenia may result in some harmful behaviors towards him/her self or other individuals in patient. Physical restrain, or chemical restrain with psychotropic drugs can be used for ensuring the safety of the patient with a tendency toward violent behavior and to prevent harm to himself and others. These restrain methods are usually applied when they showed aggressive or violent behaviors, that is after the observed warning signs or real violent behaviors.There is no system that can evaluate and notice agitation or tendency of violence before the obvious behaviors. By using a wearable sensor system to be able to measure some biological change and to evaluate of the sensors’ ability to obtain quantitative and objective data may help the health professionals to prevent the damage in advance.

**Objectives:**

The aim of this pilot study was to evaluate the changes in measurements of the four wearable sensors which applied to persons with schizophrenia.

**Methods:**

Ten patients who restrained in the observation room, selected for this pilot study. On the first day (13:00), which meets the criteria for inclusion in the study and the end of the insulation process (the COVID test result is negative), the first measurement was before the noon treatment. The patients’ second measurements were taken on the day they switched from parenteral to oral treatment. For measurement, the sensor circuits developed at the Physiological Analysis and Wearable Systems Research Laboratory of Koç University were connected to various parts of the body to collect the non-invasive data detailed below. In addition, including the clinical status of the patient in the experimentation process, and a positive-negative syndrome scale was also used. The data from the patient was obtained under the supervision of the clinical chief nurse for 10 minutes. Sensors were electrocardiogram (ECG) photoplethysmogram (PPG), seismocardiography (SCG), body temperature.

**Results:**

Since some recording errors observed in two patients’ records, the data of eight patients were evaluated. Aside from one of the eight patients evaluated, the signal deviation and strength of other patients’ data increased in general. This result imply that signal deviations and strengths may be reduced during the psychomotor agitation. These deviations may suggest that this sensor system is capable to evaluate some biological changes in patients.

**Conclusions:**

Considering the results of the pilot study, it is planned to carry out future studies with a larger sample size and longer records. With these studies, it is thought that psychomotor agitation in patients can be determined in an objective and measurable way without risk.

**Disclosure of Interest:**

None Declared

